# Regulatory role of mTORC1 signaling in osteoblasts in acute myeloid leukemia progression and steady-state hematopoiesis

**DOI:** 10.1016/j.isci.2025.114533

**Published:** 2025-12-24

**Authors:** Kazuya Fukasawa, Kazuya Tokumura, Makoto Yoshimoto, Koki Sadamori, Ioanna Mosialou, Yoshiaki Harakawa, Kazuto Isawa, Shohei Tsuji, Haruhiko Inufusa, Atsushi Hirao, Stavroula Kousteni, Eiichi Hinoi

**Affiliations:** 1 Department of Bioactive Molecules, Pharmacology, Gifu Pharmaceutical University, Gifu 501-1196, Japan; 2 Department of Physiology and Cellular Biophysics, Columbia University Medical Center, New York, NY 10032, USA; 3 Division of Anti-Oxidant Research, Life Science Research Center, Gifu University, Gifu 501-1194, Japan; 4 WPI Nano Life Science Institute (WPI-Nano LSI), Kanazawa University, Kanazawa, Ishikawa, Japan; 5 Cancer and Stem Cell Research Program, Division of Molecular Genetics, Cancer Research Institute, Kanazawa University, Kanazawa, Ishikawa, Japan; 6 United Graduate School of Drug Discovery and Medical Information Sciences, Gifu University, Gifu 501-1196, Japan; 7 Center for One Medicine Innovative Translational Research (COMIT), Division of Innovative Modality Development, Institute for Advanced Study, Gifu University, Gifu 501-1196, Japan

**Keywords:** cell biology, cancer

## Abstract

Acute myeloid leukemia (AML) is widely recognized for its intrinsic leukemic-cell-driven regulation as well as its extrinsic niche-driven regulation. Despite mounting evidence that bone-forming osteoblasts provide an endosteal niche for AML cells, the precise mechanism remains to be elucidated. The cell-autonomous mammalian target of rapamycin complex 1 (mTORC1) is involved in the onset and progression of AML. Here, we found that mTORC1 signaling was activated in the osteoblasts of an AML murine model and clinical AML specimens. Osteoblast-specific mTORC1 activation in mice promotes AML growth, whereas mTORC1 inactivation suppresses it. Interleukin-6 (IL-6) was identified through screening as a downstream factor in mTORC1-regulated AML progression. Genetic ablation of the IL-6 receptor in AML cells significantly attenuated AML growth in osteoblast-specific mTORC1-activated mice. Collectively, our results suggest that the mTORC1/IL-6 axis in osteoblastic niche non-autonomously contributes to the AML progression, suggesting a viable therapeutic target for AML.

## Highlights

• mTORC1 signaling is activated in osteoblasts of murine model and clinical specimen of AML
• mTORC1 activation in osteoblasts worsens AML progression, and vice versa
• mTORC1 activation in osteoblasts disrupts normal hematopoiesis
• mTORC1-IL-6 axis in osteoblasts contributes to AML progression

## Graphical abstract

**Figure:**
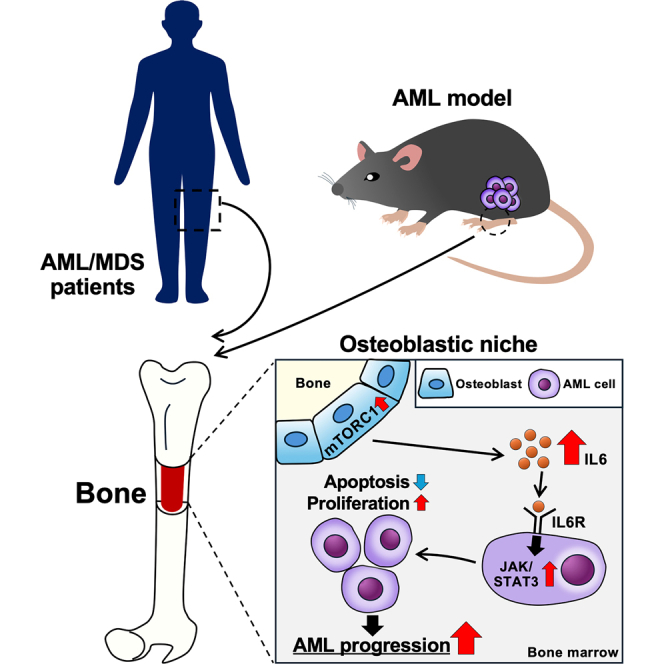

## Introduction

Acute myeloid leukemia (AML) is a heterogeneous clonal hematopoietic neoplasm that is among the most prevalent hematological malignancies in older adults, with high recurrence and mortality rates even after various treatments.^1^^,^^2^^,^^3^ The current treatment for most AML patients still largely relies on standard “7 + 3” chemotherapy and allogeneic stem cell transplantation, despite extensive research leading to the development of viable targeted agents.^4^ Myelodysplastic neoplasms (MDS), another group of heterogeneous clonal hematopoietic neoplasms, share a continuous disease spectrum with AML.^5^ Although genetic and epigenetic mutations within hematopoietic cells are well-established drivers of hematological malignancies such as AML and MDS, there is increasing evidence of the crucial role of the bone marrow (BM) microenvironment and leukemic niches in the initiation, propagation, and recurrence of leukemia, as well as for drug resistance and high relapse rates after chemotherapy in both mice and humans.^6^^,^^7^

The BM microenvironment is composed of both endosteal and vascular niches.^8^ The vascular niche primarily consists of sinusoidal endothelial cells (ECs) and pericytes, which have been reported to regulate AML growth and resistance to chemotherapy, while the endosteal niche includes osteoblasts, mesenchymal stem cells (MSCs), osteocytes, and osteoclasts.^9^^,^^10^ Bone-forming osteoblasts maintain proper bone mass, quality, and strength, coupled with bone-resorbing osteoclasts in the BM microenvironment.^11^ In addition to their role in bone homeostasis, osteoblastic lineage cells contribute to the regulation of normal hematopoiesis under steady-state conditions and are also linked to hematological malignancies such as MDS/AML in clinical settings.^12^^,^^13^^,^^14^ Accumulating evidence indicates that targeting niche cells is an effective strategy for improving MDS/AML treatment and contributes to the development of viable therapeutic strategies.^15^ However, the mechanisms governing cell-cell communication between niche cells and leukemia cells, the molecular events influencing leukemia pathogenesis, and the potential therapeutic exploitation of this interplay remain largely unexplored.

The mechanistic target of rapamycin (mTOR), a serine/threonine kinase belonging to the phosphatidylinositol 3-kinase-related kinase family, regulates various cellular processes, including growth, proliferation, differentiation, survival, and autophagy.^16^ mTOR forms two physically and functionally distinct complexes: mTOR complex 1 (mTORC1) and mTORC2.^17^ The regulatory-associated protein of mTOR (Raptor) subunit is part of the mTORC1 complex.^18^ Tuberous sclerosis complex 1 (*Tsc1*) and complex 2 (*Tsc2*), which encode hamartin and tuberin, respectively, are critical negative regulators of mTORC1 through GTPase activation of the small G-protein Ras homolog enriched in the brain (Rheb1).^19^

mTORC1 has been widely reported to be a critical pathway in cancer cells, including AML.^20^^,^^21^ Given that intrinsic (leukemic-cell-driven) mTORC1 activity is involved in the onset and progression of AML, its inhibition has been considered a potential treatment strategy; however, the clinical use of mTORC1 inhibitors has shown limited efficacy.^22^^,^^23^ In this study, we investigated whether extrinsic (niche-driven) mTORC1 activity in the BM microenvironment is involved in AML progression using an integrated cell-specific knockout strategy in combination with a well-established AML murine model, bioinformatic approaches, and clinical specimens to elucidate viable niche cell-targeting therapeutic strategies against hematological malignancies.

## Results

### mTORC1 signaling is activated in AML murine model osteoblasts

**Figure 1:**
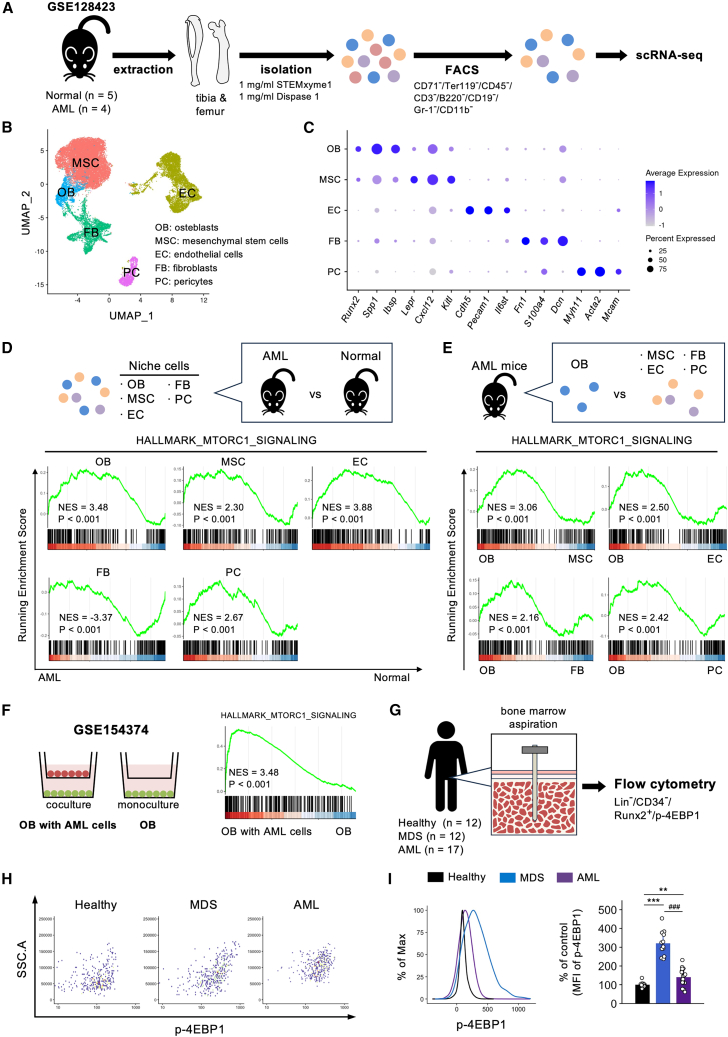
mTORC1 signaling is activated in osteoblasts of AML murine model and patients with MDS/AML (A) Schematic diagram of the scRNA-seq analysis using a *MLL-AF9* murine AML model. (B) Uniform manifold approximation and projection (UMAP) plot showing five distinct niche cell clusters identified in the bone marrow microenvironment. (C) Feature plots of canonical marker genes used to identify five cell clusters: osteoblasts (OBs; *Runx2*^*+*^, *Spp1*^*+*^, and *Ibsp*^*+*^), mesenchymal stem cells (MSCs; *Lepr*^*+*^, *Cxcl12*^*+*^, and *Kitl*^*+*^), endothelial cells (ECs; *Cdh5*^*+*^, *Pecam1*^*+*^, and *Il6st*^*+*^), fibroblasts (FBs; *Fn1*^*+*^, *S100a4*^*+*^, and *Dcn*^*+*^), and pericytes (PCs; *Myh11*^*+*^, *Acta2*^*+*^, and *Mcam*^*+*^). (D–F) GSEA results for the HALLMARK_MTORC1_SIGNALING gene set; (D) comparison across all niche cell types (OB, MSC, EC, FB, and PC) in murine AML model and normal, (E) comparison between OB and other niche cell types (MSC, EC, FB, and PC) in murine AML model, and (F) comparison between OB co-cultured with AML cells and monocultured OB. (G) Schematic diagram of bone marrow aspiration and flow cytometry. (H and I) (H) Representative flow cytometry plots and (I) MFI of phosphorylated 4E-BP1 in Lin^–^CD34^–^Runx2^+^ osteoblasts from the bone marrow of healthy, MDS, and AML (Healthy: *n* = 12, MDS: *n* = 12, AML: *n* = 17, Kruskal-Wallis test followed by pairwise Wilcoxon rank-sum tests with Benjamini-Hochberg adjustment). ∗∗*p* < 0.01, ∗∗∗*p* < 0.001, and ^###^*p* < 0.001. Error bars show the standard deviation.

In contrast to its critical intrinsic role, the extrinsic role of mTORC1 in AML pathogenesis remains unclear. To investigate the activity of mTORC1 signaling in AML niches, the single-cell RNA sequencing (scRNA-seq) dataset of a murine AML model initiated by *MLL-AF9* (a fusion gene resulting from the chromosomal translocation t^9^^,^^11^[p22;q23]) was first analyzed (Figure 1A). Five clusters were successfully identified through clustering analysis: osteoblasts (cluster 1; *Runx2*^*+*^, *Spp1*^*+*^, and *Ibsp*^*+*^), MSCs (cluster 2; *Lepr*^*+*^, *Cxcl12*^*+*^, and *Kitl*^*+*^), ECs (cluster 3; *Cdh5*^*+*^, *Pecam1*^*+*^, and *Il6st*^*+*^), fibroblasts (cluster 4; *Fn1*^*+*^, *S100a4*^*+*^, and *Dcn*^*+*^), and pericytes (cluster 5; *Myh11*^*+*^, *Acta2*^*+*^, and *Mcam*^*+*^) (Figures 1B and 1C). Gene set enrichment analysis (GSEA) revealed that the mTORC1 pathway was significantly enriched in all niche cells except for fibroblasts in AML mice compared to that in the niche cells of control mice (Figure 1D). Further analyses revealed that the mTORC1 pathway was significantly enriched in osteoblasts compared to that in MSCs, ECs, fibroblasts, and pericytes in the murine AML model (Figure 1E). In contrast, the mTORC1 pathway was found to be significantly enriched in osteoblasts in comparison to that observed in MSCs, ECs, and pericytes in control mice (Figure S1). In accordance with the results of murine scRNA-seq datasets, bulk RNA sequencing (bulk RNA-seq) analysis revealed that the mTORC1 signaling was significantly enriched in healthy-subject-derived primary human osteoblasts co-cultured with human AML cells carrying the *MLL-AF9* fusion oncogene compared to that in cultured osteoblasts alone (Figure 1F).

### mTORC1 signaling is activated in osteoblasts of patients with MDS/AML

We then attempted to demonstrate the relevance of these bioinformatics findings in clinical settings by measuring mTORC1 activity in the osteoblasts of patients with MDS/AML using flow cytometry (Figure 1G). In accordance with the results of bioinformatics analyses, the expression levels of phosphorylated 4E-BP1, downstream of mTORC1,^16^ among lineage^–^CD34^–^Runx2^+^ cells of the BM were significantly increased in patients with both MDS and AML compared to those in control participants (Figures 1H and 1I). Collectively, the bioinformatics studies of murine AML model and human cells as well as the clinical studies of patients with MDS/AML suggest that mTORC1 activity in osteoblasts might be associated with AML pathogenesis.

### mTORC1 activation in osteoblasts worsens AML progression

**Figure 2:**
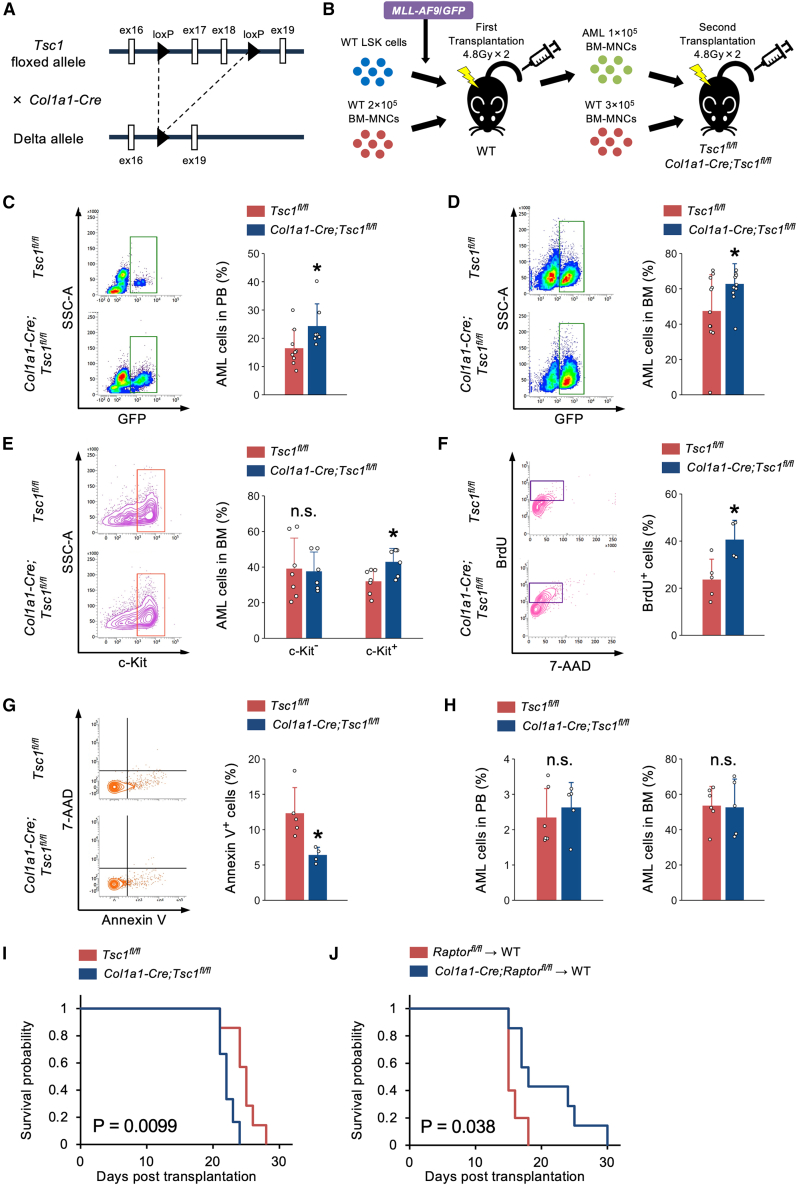
mTORC1 activation in osteoblasts accelerates AML progression (A) Schematic diagram of generation of tissue-specific *Tsc1* knockout mice. (B) Schematic diagram of generation of a *MLL-AF9* murine AML model. (C and D) Representative flow cytometry plots and percentages of GFP^+^ AML cells in the (C) peripheral blood (*n* = 7–9) and (D) bone marrow (*n* = 11) of *Tsc1*^*fl/fl*^ and *Col1a1-Cre;Tsc1*^*fl/fl*^ mice. (E–G) Representative flow cytometry plots and percentages of (E) GFP^+^c-Kit^–^ AML cells and GFP^+^c-Kit^+^ AML cells (*n* = 6–7), (F) BrdU^+^GFP^+^c-Kit^+^ AML cells (*n* = 4–5), and (G) Annexin V^+^GFP^+^c-Kit^+^ AML cells (*n* = 4–5) in the bone marrow of *Tsc1*^*fl/fl*^ and *Col1a1-Cre;Tsc1*^*fl/fl*^ mice. (H) Percentages of GFP^+^ AML cells in the peripheral blood and bone marrow of *Tsc1*^*fl/fl*^ and *Col1a1-Cre;Tsc1*^*fl/fl*^ mice 8 days after the second transplantation (*n* = 3). (I and J) Survival probabilities of (I) *Tsc1*^*fl/fl*^ and *Col1a1-Cre;Tsc1*^*fl/fl*^ AML mice (*n* = 6–7) and (J) WT AML mice transplanted with c-Kit^+^ AML cells from *Raptor*^*fl/fl*^ and *Col1a1-Cre;Raptor*^*fl/fl*^ mice (*n* = 5–7). All mice used in this study were male. n.s., not significant. ∗*p* < 0.05. Error bars show the standard deviation.

Given the possible association between cell non-autonomous mTORC1 activity and AML pathogenesis (Figure 1), we investigated the role of mTORC1 activity within niche cells in AML progression using niche-specific conditional knockout mice in combination with a well-established *MLL-AF9*-induced AML murine model that transforms hematopoietic progenitor cells into AML cells. We first induced osteoblast-specific mTORC1 activation in mice through the genetic ablation of *Tsc1*/Hamartin, a negative regulator of mTORC1,^19^ by crossing *Tsc1*-floxed mice with *Collagen type 1 alpha 1* (*Col1a1*)*-Cre* mice (Figure 2A). To create a murine AML model, we introduced the *MLL-AF9* fusion gene into lineage^–^Sca-1^+^c-Kit^+^ (LSK) cells isolated from wild-type (WT) mice using retrovirus-mediated transfer. We then transplanted these genetically modified cells into lethally irradiated syngeneic recipients, along with WT rescue cells, to generate WT-AML mice. To determine the effect of mTORC1 activation within osteoblasts on AML progression *in vivo*, we performed a second transplantation in which BM mononuclear cells (BM-MNCs) from WT-AML mice were transferred into lethally irradiated control and *Col1a1-Cre;Tsc1*^*fl/fl*^ recipient mice (Figure 2B). We first analyzed the characteristics of AML cells in osteoblast-specific mTORC1-activation mice 17 days after the second transplantation. Cytometric analyses revealed that *Col1a1-Cre;Tsc1*^*fl/fl*^ mice displayed a significantly higher proportion of AML cells in both peripheral blood and BM than control mice (Figures 2C and 2D). c-Kit marks undifferentiated AML cells in murine AML models.^31^^,^^32^ *Col1a1-Cre;Tsc1*^*fl/fl*^ mice showed a significant increase in the proportion of c-Kit^+^ AML cells but not c-Kit^–^ AML cells in the BM (Figure 2E). We then examined whether mTORC1 activation in osteoblasts affects AML cell proliferation and apoptosis. *Col1a1-Cre;Tsc1*^*fl/fl*^ mice displayed a significant increase in cell proliferation and a decrease in cell apoptosis of c-Kit^+^ AML cells in the BM compared to those in control mice (Figures 2F and 2G). On the contrary, no significant changes were seen in AML cells in the peripheral blood and BM when evaluated 8 days after the second transplantation (Figure 2H). Finally, the survival probability was significantly lower in *Col1a1-Cre;Tsc1*^*fl/fl*^ mice than in control mice following the second transplantation (Figure 2I).

### mTORC1 activation in MSC and EC does not affect AML progression

Although mTORC1 activation was highest within osteoblasts among niche cells of the murine AML model, bioinformatics analyses revealed its significant activation in other niche cells (Figure 1C). Next, we assessed whether mTORC1 activity in other niche cells could influence AML progression. We generated MSC-specific and EC-specific *Tsc1*-knockout mice, referred to as *Leptin receptor* (*LepR*)-*Cre;Tsc1*^*fl/fl*^ and *Cadherin 5* (*Cdh5*)*-CreER;Tsc1*^*fl/fl*^ mice, using either *LepR-Cre* or *Cdh5-CreERT2* mice, respectively. These mice were used to create a murine AML model via AML cell transfer, as done with *Col1a1-Cre;Tsc1*^*fl/fl*^ mice (Figures 2A and 2B). The *MLL-AF9*-induced AML model was established in *Cdh5-CreER;Tsc1*^*fl/fl*^ mice 4 weeks after five consecutive tamoxifen injections. Our findings showed that neither *LepR-Cre;Tsc1*^*fl/fl*^ mice nor *Cdh5-CreER;Tsc1*^*fl/fl*^ mice exhibited significant changes in mortality (Figures S2A and S2B). Collectively, these results indicate that while mTORC1 activation in osteoblasts exacerbates AML progression by increasing proliferation and decreasing apoptosis of undifferentiated AML cells, its activation in MSCs and ECs does not contribute to AML progression.

### mTORC1 inactivation in osteoblasts weakens AML progression

Although *Tsc1*/Hamartin is a well-known negative regulator of mTORC1, it also plays a pivotal role in cellular functions independent of its role in mTORC1 regulation.^33^ We observed exacerbated AML progression in *Col1a1-Cre;Tsc1*^*fl/fl*^ mice (Figure 2). We, therefore, investigated whether mTORC1 activity in osteoblasts indeed plays a critical role in AML pathogenesis. Raptor, a constitutively binding protein of mTORC1,^18^ is essential for mTORC1 activity. We generated osteoblast-specific mTORC1-inactivated mice, termed *Col1a1-Cre;Raptor*^*fl/fl*^ mice (Figure S3A). To determine the effect of mTORC1 inactivation in osteoblasts on AML progression *in vivo*, we performed a second transplantation where BM-MNCs from WT-AML mice were transferred into control and *Col1a1-Cre;Raptor*^*fl/fl*^ mice. Survival probability was comparable between *Col1a1-Cre;Raptor*^*fl/fl*^ and control mice (Figure S3B). Serial transplantation of *MLL-AF9*-transduced murine AML cells resulted in the enrichment of stemness and an aggressive phenotype.^34^ We then isolated AML cells from different donor mice (*Col1a1-Cre;Raptor*^*fl/fl*^ and control mice) following a second transplantation, and an equal number of c-Kit^+^ AML cells were transplanted into lethally irradiated WT mice as a third transplantation (Figure S3C). The survival probability was significantly higher in WT mice transplanted with *Col1a1-Cre;Raptor*^*fl/fl*^-derived AML cells than in WT mice transplanted with control-derived AML cells (Figure 2J). Taken together, the results of both gain-of-function and loss-of-function studies of mTORC1 activity, combined with an *MLL-AF9* murine model, suggest that mTORC1 activity in osteoblasts contributes to AML progression.

### Activation of mTORC1 in osteoblasts disrupts normal hematopoiesis

**Figure 3:**
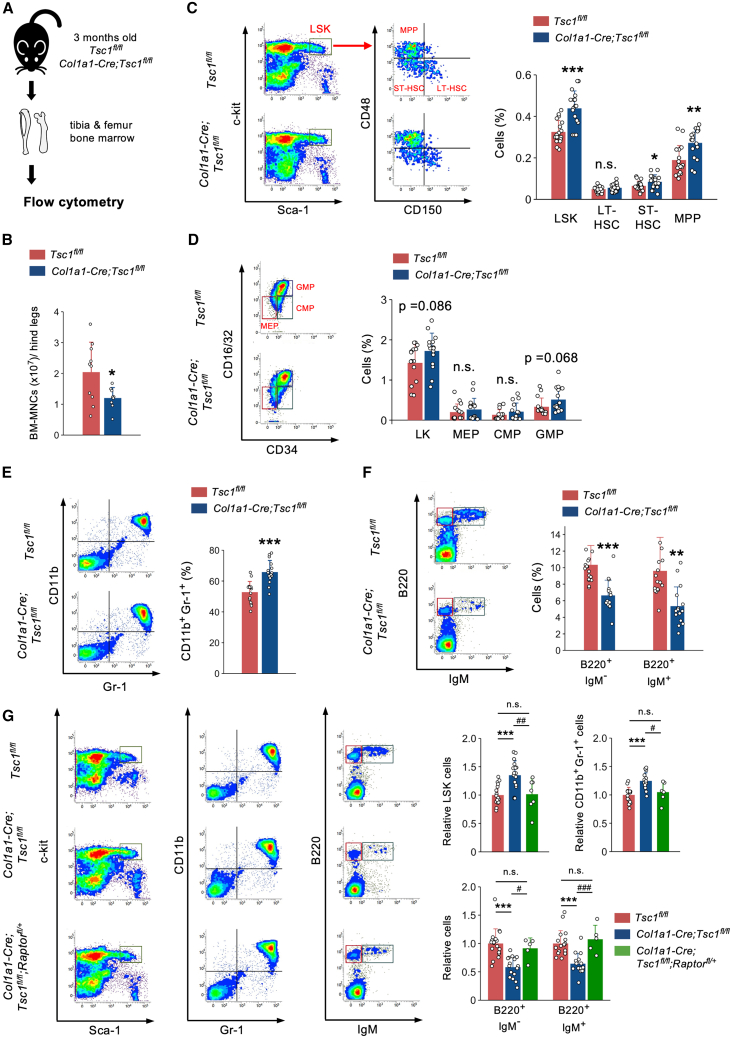
Activation of mTORC1 in osteoblasts impairs normal hematopoiesis (A) Schematic diagram of flow cytometric analysis of *Tsc1*^*fl/fl*^ and *Col1a1-Cre;Tsc1*^*fl/fl*^ mice. (B) The number of BM-MNCs in hind legs of *Tsc1*^*fl/fl*^ and *Col1a1-Cre;Tsc1*^*fl/fl*^ mice (*n* = 9–10). (C–F) Representative flow cytometry plots and percentages of (C) LSK cells and LSK subpopulations (*n* = 17–18), (D) LK cells and LK subpopulations (*n* = 14–15), (E) CD11b^+^Gr-1^+^ myeloid cells (*n* = 15–16), and (F) B220^+^IgM^–^ immature and B220^+^IgM^+^ mature B cells (*n* = 16–18) in the bone marrow of *Tsc1*^*fl/fl*^ and *Col1a1-Cre;Tsc1*^*fl/fl*^ mice. (G) Representative flow cytometry plots and fold changes in the percentage of LSK cells (*n* = 7–18), CD11b^+^Gr-1^+^ myeloid cells (*n* = 5–18), and B220^+^IgM^–^ immature and B220^+^IgM^+^ mature B cells (*n* = 5–16) in the bone marrow of *Tsc1*^*fl/fl*^, *Col1a1-Cre;Tsc1*^*fl/fl*^, and *Col1a1-Cre;Tsc1*^*fl/fl*^*;Raptor*^*fl/+*^ mice. All mice used in this study were male. n.s., not significant. ∗*p* < 0.05, ∗∗*p* < 0.01, ∗∗∗*p* < 0.001, ^#^*p* < 0.05, ^##^*p* < 0.01, and ^###^*p* < 0.001. Error bars show the standard deviation.

Next, we evaluated whether mTORC1 signaling in osteoblasts was implicated in normal hematopoiesis (Figure 3A). Under steady-state conditions, the number of BM-MNCs was significantly decreased in the long bones of *Col1a1-Cre;Tsc1*^*fl/fl*^ mice (Figure 3B). *Col1a1-Cre;Tsc1*^*fl/fl*^ mice displayed a significantly higher proportion of LSK cells along with significant increases in short-term HSCs and multipotent progenitors (MPPs) in the BM, but not long-term HSCs, compared to those in control mice (Figure 3C). In contrast, the proportions of lineage^–^Sca-1^–^c-Kit^+^ (LK) cells, megakaryocyte-erythrocyte progenitors (MEPs), and common myeloid progenitors (CMPs) in the BM were comparable between *Col1a1-Cre;Tsc1*^*fl/fl*^ and control mice, despite a trend toward an increase in the proportion of granulocyte-macrophage progenitors (GMPs) in *Col1a1-Cre;Tsc1*^*fl/fl*^ mice (Figure 3D). The proportion of CD11b^+^Gr-1^+^ myeloid cells was significantly increased in *Col1a1-Cre;Tsc1*^*fl/fl*^ mice (Figure 3E). In contrast, the proportions of both B220^+^IgM^–^ (pro- and pre-B) and B220^+^IgM^+^ (immature and mature B) lymphocytes significantly decreased in the BM of *Col1a1-Cre;Tsc1*^*fl/fl*^ mice (Figure 3F). To assess whether mTORC1 signaling was directly involved in the abnormal regulation of hematopoiesis observed in *Col1a1-Cre;Tsc1*^*fl/fl*^ mice, we generated *Col1a1-Cre;Tsc1*^*fl/fl*^ mice lacking one copy of the floxed *Raptor* allele (*Col1a1-Cre;Tsc1*^*fl/fl*^*;Raptor*^*fl/+*^). The abnormalities observed in *Col1a1-Cre;Tsc1*^*fl/fl*^ mice were significantly ameliorated in *Col1a1-Cre;Tsc1*^*fl/fl*^*;Raptor*^*fl/+*^ mice (Figure 3G). These results collectively indicate that similar to its effect on AML progression, mTORC1 activity in osteoblasts may affect steady-state hematopoiesis.

### Interleukin-6 in osteoblasts is a critical factor for AML progression

**Figure 4:**
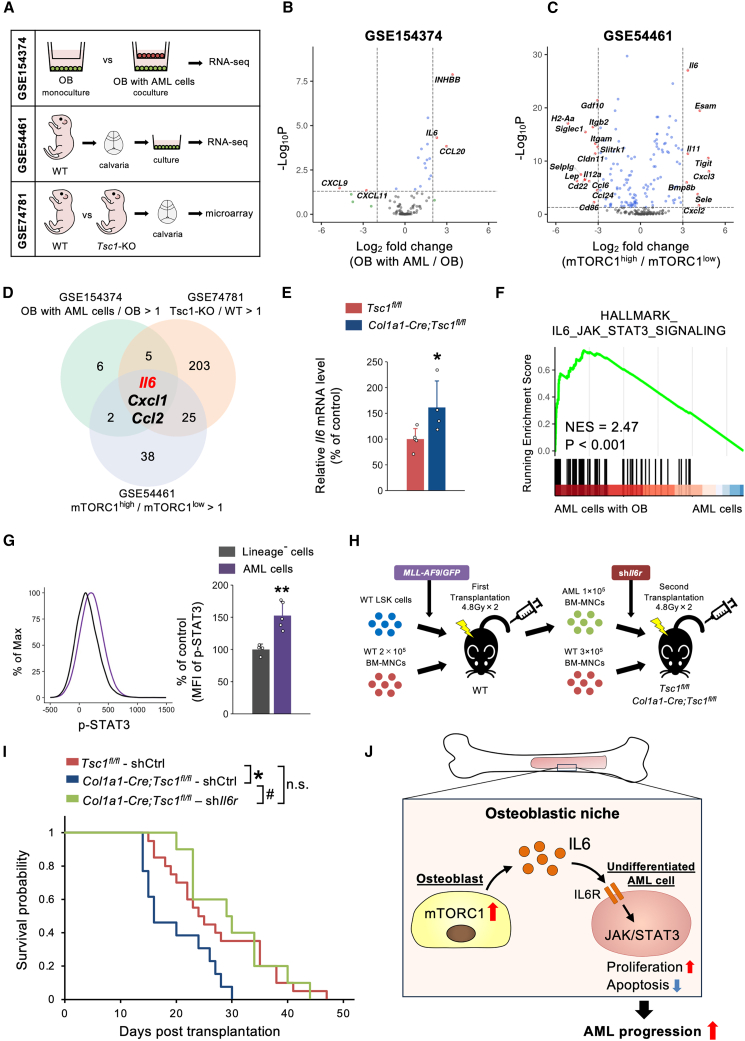
IL-6 in osteoblasts plays a crucial role for AML progression (A) Schematic diagram of three datasets used to screen for factors involved in the cell-cell interaction between mTORC1-activated osteoblasts and AML cells. (B and C) Volcano plots showing DEGs (B) in osteoblasts co-cultured with AML cells compared to monocultured osteoblasts and (C) in mTORC1^high^ osteoblasts compared to mTORC1^low^ osteoblasts. (D) Venn diagram highlighting overlapping genes identified across the three datasets. (E) *Il6* mRNA expression in calvarial osteoblasts from *Col1a1-Cre;Tsc1*^*fl/fl*^ mice (*n* = 4–5). (F) GSEA results for the HALLMARK_IL6_JAK_STAT3_SIGNALING gene set in AML cells co-cultured with osteoblasts. (G) MFI of phosphorylated STAT3 in AML cells in the murine AML model and in lineage^–^ cells from WT mice (*n* = 4–5). (H) Schematic diagram of generation of the IL-6R-knockdown *MLL-AF9* murine AML model. (I) Survival probabilities of *Tsc1*^*fl/fl*^ and *Col1a1-Cre;Tsc1*^*fl/fl*^ mice transplanted with shCtrl- or sh*Il6r*-transduced AML cells (*n* = 10–20). (J) Schematic model of the findings of this study. Osteoblastic mTORC1 signaling enhances IL-6 production, which activates JAK/STAT3 signaling to promote cell proliferation and inhibit apoptosis in undifferentiated AML cells, driving AML progression. All mice used in this study were male. n.s., not significant. ∗*p* < 0.05, ∗∗∗*p* < 0.001, and ^#^*p* < 0.05. Error bars show the standard deviation.

We aimed to identify pivotal factors in osteoblasts downstream of mTORC1 that render the AML niche permissive for AML progression. We screened three different cohorts and identified secreted and adherent factors in (1) human osteoblasts co-cultured with AML cells, (2) mTORC1-activated mature osteoblasts (derived from *Osteocalcin-Cre;Tsc1*^*fl/fl*^ mice), and (3) mTORC1^high^ murine osteoblasts (Figures 4A–4C). Among the significantly upregulated genes in the three cohorts, interleukin-6 (IL-6) was identified as a potential overlapping gene (Figure 4D). We confirmed the significant upregulation of IL-6 in calvaria osteoblasts of *Col1a1-Cre;Tsc1*^*fl/fl*^ mice, indicating a positive correlation between mTORC1 activity and IL-6 expression in osteoblasts (Figure 4E). Next, we investigated whether the mTORC1-IL-6 axis in osteoblasts could contribute to AML progression. JAK/STAT3 signaling, a critical factor downstream of the IL-6 receptor (IL-6R),^35^ was enriched in AML cells co-cultured with osteoblasts (Figure 4F). Furthermore, phosphorylation of STAT3 was significantly increased in AML cells in the AML murine model (Figure 4G). To demonstrate the pivotal role of communication between osteoblasts and AML cells via the IL-6-IL-6R axis *in vivo*, IL-6R was knocked down by shRNA in BM-MNCs from WT-AML cells and subsequently transferred into lethally irradiated *Col1a1-Cre;Tsc1*^*fl/fl*^ mice as a second transplantation (Figure 4H). Lethality was significantly ameliorated in *Col1a1-Cre;Tsc1*^*fl/fl*^ mice transplanted with IL-6R-knockdown AML cells compared to those transplanted with shControl (shCtrl) (Figure 4I). Collectively, communication between osteoblasts and AML cells through the mTORC1-IL-6 and IL-6R-JAK/STAT3 axes may contribute to the progression of AML (Figure 4J).

## Discussion

AML is widely recognized as having both cell-autonomous (intrinsic leukemic cell-driven) regulation and non-cell-autonomous (extrinsic niche-driven) regulation, raising the question of whether targeting signals and factors of niche cells may provide a more stable therapeutic target than emerging leukemic clones. Early studies showed that constitutive activation of Wnt signaling by targeting a stabilizing β-catenin mutation in osteoblasts (*Col1a1-caCtnnb1* mice) leads to the development of AML, at least in part, through the Notch ligand Jagged1.^36^ Moreover, there is growing evidence that the osteogenic niche plays an important role in the development and progression of AML.^37^ Meanwhile, intrinsic (leukemic-cell-driven) mTORC1 activity has been reported to be involved in the onset and progression of AML. For example, the deletion of *Rheb1* in a murine *MLL-AF9* model resulted in increased survival through the suppression of mTOR signaling.^38^ Deletion of *Raptor* was associated with the inhibition of leukemia in a murine AML model through apoptosis of differentiated leukemia cells, whereas transplantation of *Raptor*-deficient AML cells demonstrated that mTORC1 is critical for the initiation of leukemia, suggesting that the loss of mTORC1 supports the ability for self-renewal of leukemia stem cells.^27^ In this study, we showed that mTORC1 signaling in osteoblasts governs AML progression, revealing the alternative non-cell-autonomous (osteoblastic niche-driven) regulation of AML.

Our clinical studies conducted on patients with MDS/AML have demonstrated that mTORC1 signaling was elevated in the following order: healthy subjects, patients with AML, and patients with MDS, showing no clear stepwise increase pattern. This finding suggests that mTORC1 in osteoblasts might be already elevated from the early stages of disease progression (the MDS stage), but this does not negate our central conclusion that “mTORC1 in osteoblastic niche non-autonomously contributes to the AML progression.” Conversely, it is posited that this finding could be interpreted as underscoring the notion that abnormalities in the pathological niche emerge even before the onset of the disease, thereby providing more robust evidence for clinical relevance.

The IL-6 signaling is widely recognized as a significant pathway to various cancers, including hematological malignancies.^35^^,^^39^^,^^40^ Elevated levels of IL-6 in the BM are associated with treatment resistance and poor outcomes in both pediatric and adult AML patients.^41^^,^^42^ IL-6 signaling through the JAK/STAT3 axis is increased in blood progenitors/stem cells of high-risk adult AML patients.^43^ IL-6-induced STAT3 activity correlates with inferior survival following AML relapse. Treatment with siltuximab, an IL-6-blocking antibody, mitigated AML-induced anemia and BM failure, thereby extending overall survival in mouse models.^44^ The screening of secreted and adhesion factors in osteoblasts suggested the potential involvement of alternative candidate pathways (Figure 4). The roles of C-X-C motif ligand 1 (Cxcl1) and C-C motif chemokine ligand 2 (Ccl2) in the development of various cancers, including leukemia, have been well documented.^45^^,^^46^ However, the functional roles of these factors expressed by osteoblasts in the bone microenvironment remains to be elucidated. Although it is not possible to exclude the possibility that alternative osteoblastic factors downstream of mTORC1, such as Cxcl1 and Ccl2, could be involved in the development of AML, communication between osteoblasts and AML cells within the BM microenvironment plays a crucial role in AML progression, at least in part, through mTORC1-IL-6 axis in osteoblasts and IL-6R-JAK/STAT3 axis in AML cells.

AML is the most common type of leukemia among adults. Current standard care and therapies targeting malignant cells often fail to eliminate resistant clones or prevent the expansion of new ones.^47^ Our study reveals that the interplay between osteoblasts and AML cells through the mTORC1/IL-6-IL-6R/JAK/STAT3 axis contributes to the feedforward loop of AML progression, suggesting that modifying this axis could offer an effective strategy for disease management and relapse prevention.

### Limitations of the study

This study has some limitations. AML cells share common niches with hematopoietic cells in the BM, and the non-cell-autonomous extrinsic role of niche cells contributes to AML pathogenesis in addition to the normal hematopoiesis.^48^^,^^49^ Signals from AML cells can remodel the osteoblastic niche, leading to alterations in the BM microenvironment that selectively support the malignant clone.^50^^,^^51^ To develop viable therapeutic strategies targeting cell-cell communications between niche cells and AML cells, we should identify the pivotal candidate factors downstream of IL-6R/JAK/STAT3 signaling in AML cells that could not only stimulate mTORC1 activity in osteoblasts but also remodel the osteoblastic niche to favor AML progression.

## Resource availability

### Lead contact

Further information and requests for resources and reagents should be directed to and will be fulfilled by the lead contact, Eiichi Hinoi (hinoi-e@gifu-pu.ac.jp).

### Materials availability

This study did not generate new unique reagents.

### Data and code availability

• The GSE128423, GSE154374, GSE54461, and GSE74781 datasets are deposited in the GEO database (https://www.ncbi.nlm.nih.gov/geo/).
• This paper does not report original code.
• Any additional information required to reanalyze the data reported in this paper is available from the lead contact upon request.

## Acknowledgments

We are grateful for the technical support from the members of the Hinoi lab. This work was supported by the 10.13039/501100001691Japan Society for the Promotion of Science (20H03407 to E.H.) and 10.13039/501100001695JST 10.13039/501100025019SPRING (JPMJSP2142 to M.Y.).

## Author contributions

K.F., K.T., K.S., M.Y., I.M., and E.H. conceived the project. K.F., K.T., M.Y., K.S., I.M., Y.H., and K.I. performed the experiment and analysis. S.T., H.I., A.H., and S.K. provided critical samples and discussed the results. K.F., K.T., and E.H. wrote the manuscript.

## Declaration of interests

The authors declare no potential conflicts of interest.

## STAR★Methods

### Key resources table

**Table:** 

REAGENT or RESOURCE	SOURCE	IDENTIFIER
**Antibodies**		

Purified anti-mouse CD16/32 Antibody	BioLegend	Cat#101302 RRID: AB_312801
Fixable Viability Stain 780	BD Biosciences	Cat# 565388 RRID: AB_2869673
PE mouse anti-stat3 (pY705)	BD Biosciences	Cat# 562072 RRID: AB_10893601
APC mouse linage antibody cocktail	BD Biosciences	Cat# 51-9003632 RRID: AB_1645213
Brilliant Stain Buffer	BD Biosciences	Cat# 563794 RRID: AB_2869750
PE-Cy7 Rat Anti-Mouse Ly-6A/E	BD Biosciences	Cat #561021 RRID: AB_647253
FITC Rat Anti-Mouse CD117	BD Biosciences	Cat #553354 RRID: AB_394805
APC Rat Anti-Mouse CD117	BD Biosciences	Cat #561074 RRID: AB_398536
PE Rat Anti-Mouse CD150	BD Biosciences	Cat #562651 RRID: AB_2737705
BV421 Hamster Anti-Mouse CD48	BD Biosciences	Cat #747718 RRID: AB_2872197
PE Rat Anti-Mouse CD16/CD32	BD Biosciences	Cat #553145 RRID: AB_394660
BV421 Rat Anti-Mouse CD34	BD Biosciences	Cat #562608 RRID: AB_11154576
BV421 Rat Anti-Mouse CD127	BD Biosciences	Cat #566300 RRID: AB_2737917
APC Rat Anti-Mouse CD45R/B220	BD Biosciences	Cat #553092 RRID: AB_398531
BV421 Rat Anti-Mouse IgM	BD Biosciences	Cat #562595 RRID: AB_2737671
APC anti-mouse/human CD11b Antibody	BioLegend	Cat #101211 RRID: AB_312794
BV421 Rat Anti-Mouse Ly-6G and Ly-6C	BD Biosciences	Cat #562709 RRID: AB_2737736
Pacific Blue anti-human Lineage Cocktail	BioLegend	Cat #348805 RRID: AB_2889063
Alexa Fluor 700 Mouse anti-Human CD34	BD Biosciences	Cat #561440 RRID: AB_10715443
RUNX2 Antibody	Santa Cruz	Cat #sc-10758 RRID: AB_2184247
PE Mouse Anti- BrdU	BD Biosciences	Cat #556029 RRID: AB_396305
PE Annexin V	BD Biosciences	Cat #556421 RRID: AB_2869071
7-AAD	BD Biosciences	Cat #559925 RRID: AB_2869266

**Bacterial and virus strains**		

pLKO.1 puro	Addgene	Cat#8453
Retrovirus MLL-AF9-ires-eGFP	Hirao lab, Kanazawa University, Japan	N/A
pLKO.1-shIl6r	Sigma-Aldrich	Cat#TRCN0000305257

**Biological samples**		

Human BM aspirates and biopsies (MDS/AML patients)	Myelodysplastic Syndromes Center at New York Presbyterian, Columbia University Medical Center	IRB protocols: AAAF4107
Healthy BM aspirates/biopsies	Department of Orthopedic Surgery, Columbia University	IRB protocols : AAAR3184

**Chemicals, peptides, and recombinant proteins**		

Recombinant human Thrombopoietin	PeproTech	Cat GMP300-18-50UG
Recombinant mouse SCF	FUJIFILM Wako Pure Chemical	Cat 197-12711
Recombinant mouse IL-3	FUJIFILM Wako Pure Chemical	Cat 097-06131
Polybrene	nacalai tesque	Cat 12996-81
Puromycin	FUJIFILM Wako Pure Chemical	Cat 166-23153
NH4Cl (Ammonium chloride)	FUJIFILM Wako Pure Chemical	Cat 017-02995
Paraformaldehyde (PFA)	nacalai tesque	Cat 02890-45
Polyoxyethylene^10^ Octylphenyl Ether	FUJIFILM Wako Pure Chemical	Cat 168-11805
S-Clone SF-03 medium	Sanko Junyaku	Cat #1303
RPMI-1640 medium	Sigma-Aldrich	Cat R8758-500ML
Fetal Bovine Serum (FBS)	Hyclone	Cat SH30396.03
PBS tablets	Medicago	Cat 09-9400-100
Tamoxifen	Sigma-Aldrich	Cat T5648-5G
BrdU	FUJIFILM Wako Pure Chemical	Cat 027-15561
2NA (EDTA・2Na)	DOJINDO	Cat 345-01865
MiniCollect® II Tube EDTA-2K	Greiner Bio-One	Cat 450532

**Critical commercial assays**		

Quick Taq HS Dyemix	TOYOBO	Cat DTM-101x10
Annexin V Binding Buffer	BD Biosciences	Cat 556454
One Step PrimeScript™ RT-PCR Kit (Perfect Real Time)	TaKaRa	Cat#RR064A

**Deposited data**		

scRNA-seq dataset	NCBI GEO	GSE128423
Bulk RNA-seq dataset	NCBI GEO	GSE54461
Bulk RNA-seq dataset	NCBI GEO	GSE154374
Microarray dataset	NCBI GEO	GSE74781

**Experimental models: Organisms/strains**		

*Col1a1-Cre* mice	Decquin *et al*^25^.	N/A
*LepR-Cre* mice	Jackson Laboratory	# 008320
*Cdh5-CreERT2* mice	Wang *et al*^26^.	N/A
*Tsc1*^*fl/fl*^ mice	Jackson Laboratory	#005680
*Raptor*^*fl/fl*^ mice	Jackson Laboratory	#013188
C57BL/6J mice	Japan SLC	# 000664

**Oligonucleotides**		

Primers	This paper	Tables S1 and S2

**Software and algorithms**		

Rstudio	Posit	https://posit.co/download/rstudio-desktop/
DESeq2	Bioconductor	https://bioconductor.org/packages/release/bioc/html/DESeq2.html
Seurat	Satija Lab	https://satijalab.org/seurat/
clusterProfiler	Bioconductor	https://bioconductor.org/packages/clusterProfiler
msigdbr	CRAN	https://cran.r-project.org/web/packages/msigdbr/index.html
GSVA	Bioconductor	https://bioconductor.org/packages/GSVA
presto	GitHub	https://github.com/immunogenomics/presto
enrichplot	Bioconductor	https://bioconductor.org/packages/enrichplot
EnhancedVolcano	Bioconductor	https://bioconductor.org/packages/EnhancedVolcano
flowCore	Bioconductor	https://www.bioconductor.org/packages/release/bioc/html/flowCore.html
survival	CRAN	https://cran.r-project.org/package=survival
SRA Toolkit	GitHub	https://github.com/ncbi/sra-tools
STAR	GitHub	https://github.com/alexdobin/STAR
Trim Galore	Babraham Bioinformatics	https://www.bioinformatics.babraham.ac.uk/projects/trim_galore/
FastQC	Babraham Bioinformatics	https://www.bioinformatics.babraham.ac.uk/projects/fastqc/
ggplot2	CRAN	https://cran.r-project.org/web/packages/ggplot2/index.html
Excel	Microsoft	https://www.microsoft.com/en-us/microsoft-365/excel
featureCounts	Subread package	http://subread.sourceforge.net/

**Other**		

MX3000P qPCR system	Agilent Technologies	Cat#401511
CytoFLEX S flow cytometer	Beckman Coulter	Cat#B75442
FACSVerse	BD Biosciences	Cat#651155

### Experimental model and study participant details

#### Mice

*Tsc1*^*fl/fl*^ and *Raptor*^*fl/fl*^ mice were crossed with either *Collagen type 1 alpha 1* (*Col1a1*)*-Cre*, *Leptin receptor* (*LepR*)-*Cre*, or *Cadherin 5* (*Cdh5*)*-CreERT2* mice.^24^^,^^25^^,^^26^ These mutant mice were backcrossed more than five generations with C57BL/6J mice. For inducible Cre-mediated recombination, mice were treated intraperitoneally with tamoxifen at 100 mg/kg/day for 5 consecutive days, starting at 8 weeks of age. Genotyping was performed by PCR using tail genomic DNA with specific primers listed in Table S1. Mice were bred under standard animal housing conditions at 23°C ± 1°C with a relative humidity of 55% and a light/dark cycle of 12 h, with free access to food and water. All mice used as transplantation recipients in this study were 8- to 12-week-old. The study protocol meets the guidelines of the Japanese Pharmacological Society and was approved by the Committee for the Ethical Use of Experimental Animals at Gifu Pharmaceutical University and Gifu University (Approval Numbers AG-P-N-20220088 and 2024-012R1). The number of animals used per experiment is stated in the figure legends.

#### Murine AML model

Murine AML model was generated.^27^ Lineage^–^Sca-1^+^c-Kit^+^ (LSK) cells isolated from wild-type (WT) mice were cultured overnight in serum-free S-Clone SF-03 medium (Sanko Junyaku, Tokyo, Japan) supplemented with 100 ng/mL recombinant human thrombopoietin (PeproTech, Cranbury, NJ, USA) plus 100 ng/ml rmSCF (FUJIFILM Wako Pure Chemical, Osaka, Japan). Cells were infected with retroviruses carrying *MLL-AF9*-ires-*eGFP* and then transplanted into lethally irradiated syngeneic mice, along with 2 × 10^5^ normal BM mononuclear cells (BM-MNCs). After the appearance of AML symptoms, 1 × 10^5^ BM-MNCs from the AML mice were transplanted intravenously into lethally irradiated syngeneic recipients, along with 3 × 10^5^ normal BM-MNCs. For the serial transplantation, 5 × 10^3^ c-kit^+^ AML cells sorted from the BM of *Raptor*^*fl/fl*^ or *Col1a1-Raptor*^*fl/fl*^ mice with AML were transplanted into lethally irradiated syngeneic WT mice, along with 3 × 10^5^ normal BM-MNCs. For the *Il6r* knockdown of AML cells, BM-MNCs from the AML mice were transduced with lentivirus for 24 hours in RPMI 1640 medium supplemented with 10% fetal bovine serum (FBS) and 10 ng/mL rmIL-3 (FUJIFILM Wako Pure Chemical), in the presence of 8 μg/mL polybrene. Cells were then subjected to selection by culture with 2 μg/mL puromycin for 3 days before usage for experiments. Control shRNA vector (pLKO.1 puro #8453, deposited by Bob Weinberg) was obtained from Addgene (Cambridge MA, USA). *Il6r* shRNA vector (pLKO.1.sh*Il6r* #TRCN0000305257) was purchased from Sigma-Aldrich (St. Louis, MO, USA).

### Method details

#### Quantitative real-time-PCR

Total RNA was extracted from the calvaria of neonatal mice, followed by one-step real-time quantitative PCR on an MX3000P instrument (Agilent, Santa Clara, CA, USA) by using specific primers (Table S2). Expression levels of the genes examined were normalized by using the *Gapdh* expression levels as an internal control for each sample.

#### Flow cytometry

BM cells were isolated by flushing the long bones with phosphate-buffered saline (PBS) containing 2% FBS. Peripheral blood was collected in K2 EDTA-coated capillaries from the retro-orbital vein. Red blood cells were removed by soaking with 0.15 M NH_4_Cl at room temperature for 5 min. Cells were then pre-incubated with anti-mouse CD16/32 antibody at 4°C for 20 min to block non-specific Fc receptor binding, followed by 30 min incubation with cocktails of antibodies at 4°C. For the cell cycle assay, BM cells were harvested 1 hour after intraperitoneal injection of BrdU (100 mg/kg) and subsequently incubated with antibody cocktails at 4°C. For the apoptosis assay, cells were incubated with fluorescence-conjugated Annexin V and 7-AAD in Annexin V binding buffer (BD Biosciences, San Jose, California, USA). For phosphorylated STAT3 staining, AML cells were incubated with Fixable Viability Stain 780, and subsequently fixation with 4% paraformaldehyde for 15 min at 37°C, followed by permeabilization in 0.5% Polyoxyethylene^10^ Octylphenyl Ether (TritonX-100) for 30 min at 4°C. AML cells were then subjected to incubation with the antibody mixture (anti-mouse CD16/32 antibody and anti-mouse phosphorylated STAT3 (pY705) antibody) for 45 min at 4°C. Details of the reagents used for flow cytometric analysis are provided in the Table S3. Samples were analyzed using the FACSVerse (BD Biosciences, San Jose, CA, USA) and CytoFLEXS (Beckman Coulter, Brea, CA, USA). Mean fluorescence intensity (MFI) was quantified using the “flowCore” package in R (version 2.19.0).

#### Surgical specimens

BM aspirate samples and bone biopsies from male and female patients with MDS and AML were obtained from an Institutional Review Board (IRB)–approved tissue repository at the Myelodysplastic Syndromes Center at New York Presbyterian–Columbia University Medical Center^28^ (Table S4). Three to 10 mL of BM aspirate was collected from the iliac crest of the back of the hip bone. Healthy BM aspirates and bone biopsies were obtained from the Orthopedic Surgery Department at Columbia University, in collaboration with Dr. R. Shah. Healthy patients who had a planned elective hip or knee surgery were asked about their participation in the study. All studies were approved by the Columbia University Medical Center IRB (Protocol Numbers AAAR3184 and AAAF4107), and informed written consent was obtained from all participants. Research was conducted in compliance with the declaration of Helsinki for collection and use of sample materials in research protocols, and in compliance with IRB regulations.

#### Single cell RNA sequencing (scRNA-seq) data analysis

We obtained the scRNA-seq data set (GSE128423)^29^ from the Gene Expression Omnibus (GEO) (https://www.ncbi.nlm.nih.gov/geo/). For the analysis, we used data from five control mice and four mice with *MLL-AF9*. The data were analyzed using the “Seurat” package (version 5.1.0) of the R software (version 4.4.0). First, scRNA-seq data were read with the Read10X function. In the preprocessing, low-quality cells with fewer than 200 or more than 4000 expressed genes were removed with the subset function. Normalization was performed with the SCTransform function, excluding the mitochondrial mapping percentage and using the glmGamPoi method. Batch effects across the nine samples were corrected using the IntegrateData function. Following these steps, a total of 27,635 cells (Ctrl: n = 12,799; *MLL-AF9*: n = 14,836) were used for subsequent bioinformatic analysis. Differentially expressed genes (DEGs) were identified using the Wilcoxon rank-sum test implemented in the “presto” package (version 1.0.0). Gene set enrichment analysis (GSEA) was conducted with the “clusterProfiler” package (version 4.12.0), utilizing gene sets obtained through the “msigdbr” package (version 7.5.1). Visualization of the GSEA results was performed using the “enrichplot” package (version 1.24.0).

#### Bulk RNA-sequencing (bulk RNA-seq) and microarray data analysis

We obtained the bulk RNA-seq datasets (GSE154374 and GSE54461)^28^ and microarray dataset (GSE74781)^30^ from the GEO. Fastq files were downloaded using the “SRA Toolkit” (ver 3.0.10). Trimming of raw reads was performed with “Trim Galore” (version 0.6.6), followed by quality check using “FastQC” (version 0.12.1). Reads were mapped to the hg38 human genome assembly or mm39 mouse genome assembly using “STAR” (version 2.7.8a) respectively. Gene expression levels were quantified from the resulting BAM files using “featureCounts” (version 2.0.2). DEGs were identified using the “DESeq2” package (version 1.44.0) in R software. The volcano plot was generated using the “EnhancedVolcano” package (version 1.22.0). GSEA was conducted with the “clusterProfiler” package, utilizing gene sets obtained through the “msigdbr” package. Visualization of the GSEA results was performed using the “enrichplot” package. For the GSE54461 dataset, we performed single sample GSEA using the “GSVA” package (version 1.50.5) with the “HALLMARK_MTORC1_SIGNALING” gene set (mTORC1^high^: n = 13, mTORC1^low^: n = 14). To investigate factors involved in cell-cell interactions between osteoblasts and AML cells downstream of mTORC1 signaling in osteoblasts, we analyzed the expression of genes in the “KEGG Cell adhesion molecules” and “KEGG Cytokine-cytokine receptor interaction” pathways (https://www.genome.jp/) across three datasets: GSE154374, GSE54461 and GSE74781.

### Quantification and statistical analysis

#### Statistical analysis

Unless otherwise specified, Student’s t-test and one-way ANOVA followed by Tukey–Kramer test were used to calculate statistical significance. Survival analysis was carried out using the log-rank test in the “survival” package (version 3.5-5) in R software (version 4.4.0). Graphs were made using Rstudio and Excel. Throughout this study, P < 0.05 was considered statistically significant.
