# Supplementary material for: Regulatory role of mTORC1 signaling in osteoblasts in acute myeloid leukemia progression and steady-state hematopoiesis
Source: iScience. 2025 Dec 24;29(1):114533. doi: 10.1016/j.isci.2025.114533 (PMC12808893; doi:10.1016/j.isci.2025.114533)
Supplement: Document S1. Figures S1–S3 and Tables S1–S4 [file mmc1.pdf]

iScience, Volume *29*

Supplemental information

Regulatory role of mTORC1 signaling in osteoblasts in acute myeloid leukemia progression and steady-state hematopoiesis

Kazuya Fukasawa, Kazuya Tokumura, Makoto Yoshimoto, Koki Sadamori, Ioanna Mosialou, Yoshiaki Harakawa, Kazuto Isawa, Shohei Tsuji, Haruhiko Inufusa, Atsushi Hirao, Stavroula Kousteni, and Eiichi Hinoi

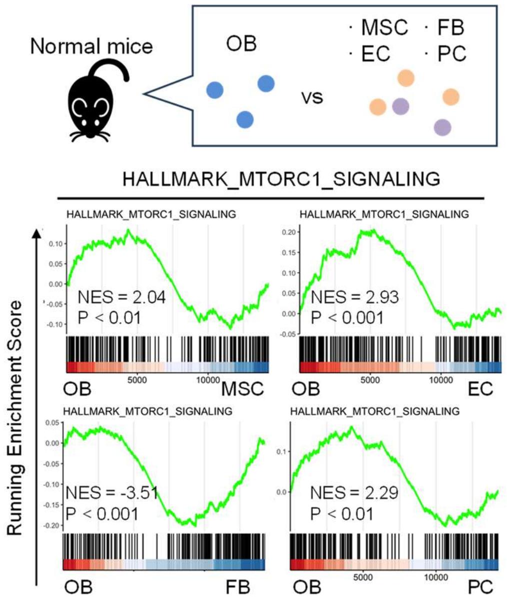

Supplementary Figure 1. GSEA results for the HALLMARK_MTORC1_SIGNALING gene set, related to Figure 1. Comparison between OB and other niche cell types (MSC, EC, FB, PC) in control mice.

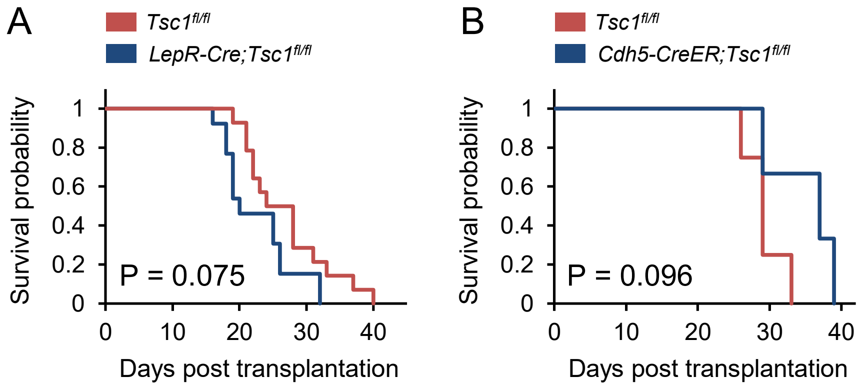

Supplementary Figure 2. Analysis of *LepR-Cre;Tsc1$^{fl/fl}$* and *Cdh5-CreER;Tsc1$^{fl/fl}$* AML mice, related to Figure 2. Survival probabilities of **(A)** *Tsc1$^{fl/fl}$* and *LepR-Cre;Tsc1$^{fl/fl}$* AML mice (n = 13-14) and **(B)** *Tsc1$^{fl/fl}$* and *Cdh5-CreER;Tsc1$^{fl/fl}$* AML mice (n = 3-4). All mice used in this study were male.

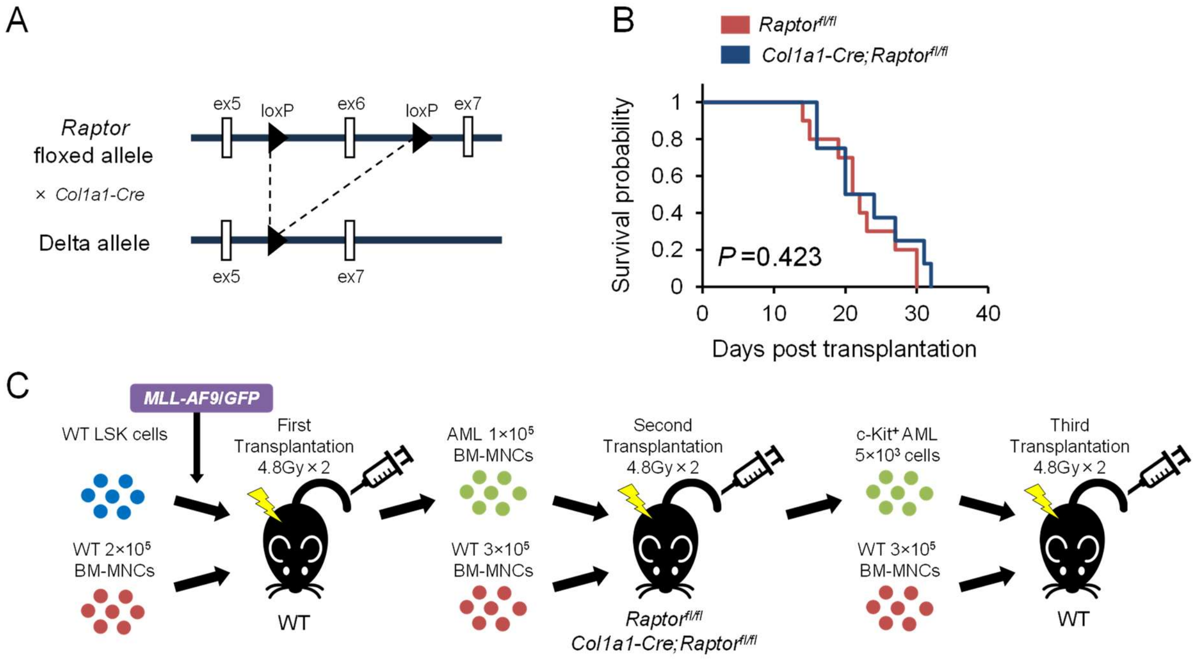

Supplementary Figure 3. Analysis of *Col1a1-Cre;Raptor$^{fl/fl}$* AML mice, related to Figure 2. **(A)** Schematic diagram of generation of tissue-specific *Raptor* knockout mice. (**B)** Survival probabilities of *Raptor$^{fl/fl}$* and *Col1a1-Cre;Raptor$^{fl/fl}$* AML mice (n = 8-10). (**C)** Schematic diagram of the serial transplantation of *MLL-AF9*-transduced murine AML cells. All mice used in this study were male.

Supplementary Table 1. List of primers used for genotyping.

Gene	Forward (5'-3')	Reverse (5'-3')
Col1a1 -Cre	GTTCCTCCCAGCTCTCCATCAAG	TGTTTAGCTGGCCCAAATGTTGCTG
Cdh5-CreER	TCCTGATGGTGCCTATCCTC	CCTGTTTTGCACGTTCACCG
LepR-Cre	CACGACCAAGTGACAGCAAT	GACAGGCTCTACTGGAATGGA
Tsc1-flox	AGGAGGCCTCTTCTGCTACCACTTTTG ATG	GAAGGCAGCTCCGACCATGAAGTGCTG TGT
Raptor-flox	TCCAAGGGAATGTATGTGGGTG	GGGTACAGTATGTCAGCACAG
Cre-control	TTACGTCCATCGTGGACAGC	TGGGCTGGGTGTTAGCCTTA

Supplementary Table 2. List of primers used for real-time PCR.

Gene	Forward (5'-3')	Reverse (5'-3')
Il6	CACCAAGAACGATAGTCAATTCCA	TCACCAGCATCAGTCCCAAG
Gapdh	AGGTCGGTGTGAACGGATTTG	TGTAGACCATGTAGTTGAGGTCA

Supplementary Table 3. List of reagents used for flow cytometric analysis

Reagent	Dilution	Source	Catalog #
Purified anti-mouse CD16/32 Antibody	1:50	BioLegend	#101301
APC Mouse Lineage Antibody Cocktail	1:10	BD Biosciences	#558074
PE-Cy7 Rat Anti-Mouse Ly-6A/E	1:50	BD Biosciences	#561021
FITC Rat Anti-Mouse CD117	1:50	BD Biosciences	#553354
APC Rat Anti-Mouse CD117	1:50	BD Biosciences	#561074
PE Rat Anti-Mouse CD150	1:50	BD Biosciences	#562651
BV421 Hamster Anti-Mouse CD48	1:50	BD Biosciences	#747718
PE Rat Anti-Mouse CD16/CD32	1:50	BD Biosciences	#553145
BV421 Rat Anti-Mouse CD34	1:50	BD Biosciences	#562608
BV421 Rat Anti-Mouse CD127	1:50	BD Biosciences	#566300
APC Rat Anti-Mouse CD45R/B220	1:50	BD Biosciences	#553092
BV421 Rat Anti-Mouse IgM	1:50	BD Biosciences	#562595
APC anti-mouse/human CD11b Antibody	1:50	BioLegend	#101211
BV421 Rat Anti-Mouse Ly-6G and Ly-6C	1:50	BD Biosciences	#562709
Pacific Blue anti-human Lineage Cocktail	1:25	BioLegend	#348805
Alexa Fluor 700 Mouse anti-Human CD34	1:100	BD Biosciences	#561440
RUNX2 Antibody	1:100	Santa Cruz	#sc-10758
PE Mouse Anti-BrdU	1:50	BD Biosciences	#556029
PE Annexin V	1:50	BD Biosciences	#556421
7-AAD	1:500	BD Biosciences	#559925
Fixable Viability Stain 780	1:1000	BD Biosciences	#565388
PE Mouse Anti-STAT3 (pY705)	1:50	BD Biosciences	#562072

Supplementary Table 4. Clinical characterization of AML and MDS patients, as well as healthy subjects, used in this study.

ID	Origin	Category	IPSS-R	Disease	Age	Sex
RS136	CUMC	Healthy	N/A	N/A	58	Male
RS137	CUMC	Healthy	N/A	N/A	53	Female
RS141	CUMC	Healthy	N/A	N/A	78	Male
RS149	CUMC	Healthy	N/A	N/A	78	Male
RS151	CUMC	Healthy	N/A	N/A	62	Female
RS155	CUMC	Healthy	N/A	N/A	73	Female
RS161	CUMC	Healthy	N/A	N/A	61	Female
RS162	CUMC	Healthy	N/A	N/A	56	Female
RS171	CUMC	Healthy	N/A	N/A	57	Male
RS172	CUMC	Healthy	N/A	N/A	68	Female
RS175	CUMC	Healthy	N/A	N/A	65	Male
RS176	CUMC	Healthy	N/A	N/A	60	Male
11383	CUMC	MDS	Low	MDS	73	Female
11384	CUMC	MDS	Low	MDS	69	Female
11386	CUMC	MDS	Very low	MDS	84	Female
11387	CUMC	MDS	Intermediate	MDS	73	Male
11388	CUMC	MDS	Very low	MDS	73	Female
11389	CUMC	MDS	N/A	MDS	71	Female
11392	CUMC	MDS	Intermediate	MDS	71	Male
11395	CUMC	MDS	Very low	MDS	76	Female
11396	CUMC	MDS	Low	MDS	66	Male
11397	CUMC	MDS	Intermediate	MDS	82	Male
11398	CUMC	MDS	Intermediate	MDS	72	Female
11399	CUMC	MDS	Very high	MDS	75	Female
AlCa	CUMC	de novo AML	N/A	AML	N/A	N/A
AnCe	CUMC	de novo AML	N/A	AML	N/A	N/A
BeKa	CUMC	de novo AML	N/A	AML	N/A	N/A
DeHa	CUMC	de novo AML	N/A	AML	N/A	N/A
EdSc	CUMC	de novo AML	N/A	AML	N/A	N/A
FeRo	CUMC	de novo AML	N/A	AML	N/A	N/A
FrCh	CUMC	de novo AML	N/A	AML	N/A	N/A
GeLe	CUMC	de novo AML	N/A	AML	N/A	N/A
GeMo	CUMC	de novo AML	N/A	AML	N/A	N/A
HeCr	CUMC	de novo AML	N/A	AML	N/A	N/A
IrGo2	CUMC	de novo AML	N/A	AML	N/A	N/A
LoMe	CUMC	de novo AML	N/A	AML	N/A	N/A
MiKe	CUMC	de novo AML	N/A	AML	N/A	N/A
PhMe	CUMC	de novo AML	N/A	AML	N/A	N/A
RoCo	CUMC	de novo AML	N/A	AML	N/A	N/A
ThOr	CUMC	de novo AML	N/A	AML	N/A	N/A
YoCh	CUMC	de novo AML	N/A	AML	N/A	N/A
